# Perforated Duodenal Ulcer 24 Years After Roux-en-Y Gastric Bypass: A Rare Presentation With Pneumoperitoneum

**DOI:** 10.7759/cureus.82107

**Published:** 2025-04-11

**Authors:** Amber Chen-Goodspeed, Nicholas Jonas, Stephen Merola, Jason Sample

**Affiliations:** 1 Department of Surgery, NewYork-Presbyterian Queens Hospital, New York City, USA; 2 Department of General Surgery, NewYork-Presbyterian Queens Hospital, New York City, USA; 3 Department of Clinical Surgery, NewYork-Presbyterian/Weill Cornell Medical Center, New York City, USA

**Keywords:** bariatric, bariatric follow up, bariatric surgery complications, duodenal perforation, duodenal ulcers, roux-en-y gastric bypass (rygb), rygb complications, rygb surgery

## Abstract

Perforated duodenal ulcers post Roux-en-Y gastric bypass (RYGB) are rare, with fewer than 30 documented cases. Diagnosis can be challenging due to the absence of pneumoperitoneum on cross-sectional imaging, seen in only five published cases.

A 67-year-old female patient, 24 years post RYGB, presented with diffuse abdominal pain, fever, hypotension, and tachycardia following recent nonsteroidal anti-inflammatory drugs (NSAIDs) use. Laboratory findings showed leukopenia and elevated lipase. Computed tomography (CT) imaging revealed pneumoperitoneum, prompting emergent exploratory laparotomy. A 0.5 cm duodenal perforation was identified and repaired with omental plication. Postoperatively, the patient developed bacteremia and intra-abdominal abscess requiring prolonged antibiotics and percutaneous drainage. Empiric *Helicobacter pylori *(*H. pylori*) treatment and lifelong proton pump inhibitors (PPI) were initiated.

No standard treatment exists for post-RYGB duodenal perforation, though omental patch plication is commonly performed. Some advocate for complete gastrectomy to prevent ulcer formation; however, risks of doing so include dysmotility, bacterial overgrowth, and recurrent ulceration. Theories regarding the etiology of these ulcers include persistent acid production in the remnant stomach, *H. pylori* infection, and potential NSAID-related effects.

Lifelong PPI use, clinical monitoring for recurrent ulcers, and early endoscopy in symptomatic patients constitute long-term patient management. While routine surveillance endoscopy is not standard, it may be considered in high-risk patients such as those with poor nutrition, ulceration while on PPI, or current smokers.

## Introduction

The Roux-en-Y gastric bypass (RYGB) is one of the most commonly performed bariatric surgeries in the United States. This operation involves dividing the stomach to create a small separate gastric pouch and connecting this pouch directly to the jejunum, bypassing the duodenum and a portion of the jejunum. This reconfiguration leads to decreased caloric and nutritional absorption and induces early satiety due to reduced stomach size. The remainder of the stomach remains anatomically attached to the duodenum, past which a jejunojejunal anastomosis is created, ensuring that bile and pancreatic enzymes can enter the digestive tract. 

Perforated duodenal ulcers are a rare but serious complication after RYGB, with fewer than 30 documented cases. Diagnosing this complication can be exceedingly difficult as, unlike most perforated viscus cases, it may present without pneumoperitoneum on cross-sectional imaging. Among the reported cases, only five demonstrated pneumoperitoneum. 

Many theories exist regarding the development of these ulcers, which include excess acid production from the gastric remnant, *Helicobacter pylori* (*H. pylori*) colonization, and nonsteroidal anti-inflammatory drugs (NSAIDs)-related effects [1-4]. These etiologies are similar to those seen and studied in peptic ulcer development [5]. While the connection between NSAIDs and peptic and marginal ulcer formation is well established, no current studies have specifically examined the relationship between NSAID use and post-RYGB duodenal ulcer formation [1, 6]. The rarity of this complication likely contributes to the lack of dedicated research.

This report aims to describe the diagnostic challenges and management considerations associated with perforated duodenal ulcers following RYGB. By presenting this case, we aim to highlight key clinical findings, potential risk factors, current treatments, and long-term surveillance options for this rare complication.

This article was previously presented as a meeting abstract at the 2024 American Society for Metabolic and Bariatric Surgery (ASMBS) Annual Scientific Meeting on June 16, 2024.

## Case presentation

A 67-year-old female patient, with a medical history significant for RYGB 24 years prior and exploratory laparotomy for small bowel obstruction 11 years prior, presented to the emergency department endorsing one day of diffuse abdominal pain. The patient had a musculoskeletal injury recently, which she managed with frequent NSAID use. Upon arrival, her vital signs were notable for fever with a temperature of 38.1°C, tachycardia to the low 100 beats per minute, and hypotension with a systolic blood pressure of 80+ mmHg. On physical examination, the patient's abdomen was distended, diffusely tender to light palpation with voluntary guarding. The patient's laboratory values were notable for significant leukopenia with a white blood cell count (WBC) of 1.9 x 10^3/uL and a mildly elevated lipase of 133 U/L. 

**Figure 1:**
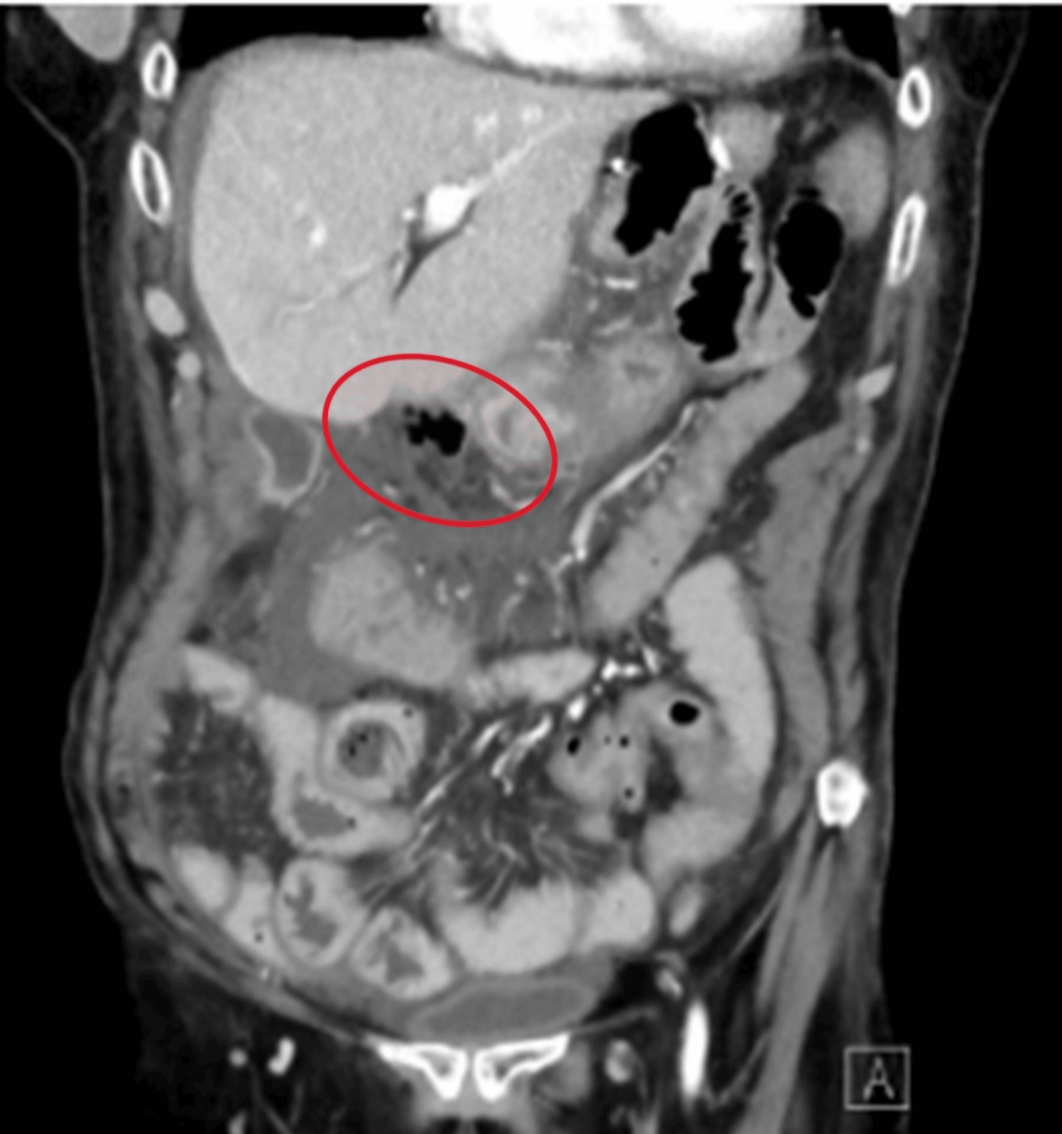
A CT of the abdomen and pelvis with the presence of free air in the abdomen near the duodenum

Computed tomography (CT) scan of the abdomen and pelvis with intravenous contrast demonstrated small volume pneumoperitoneum along the gastrocolic ligament concerning for a viscus perforation, diffuse periportal and gallbladder wall edema, and moderate perihepatic ascites tracking to the right hemipelvis (Figure 1).

**Figure 2:**
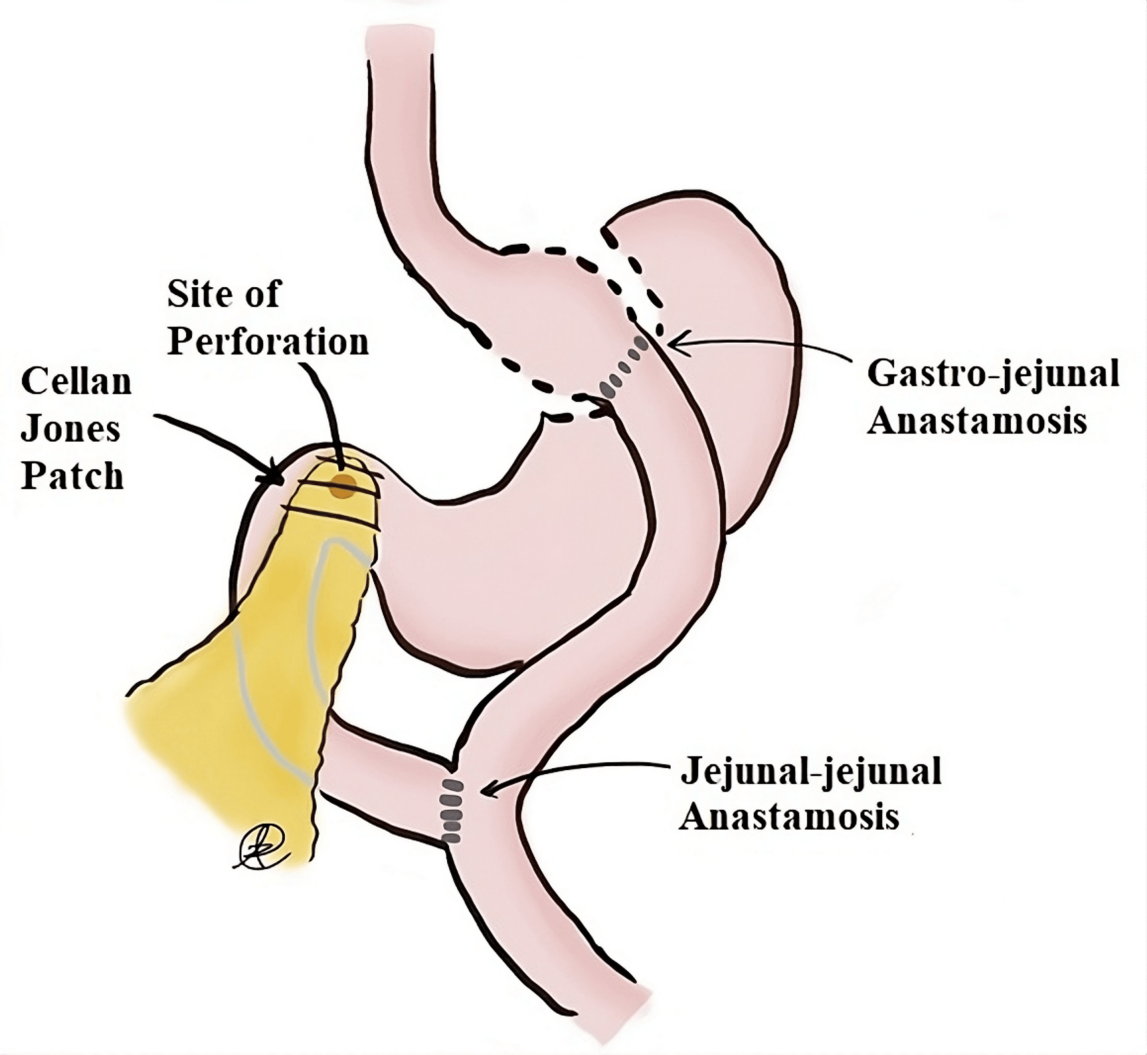
Schematic of site of duodenal perforation, anatomy, and repair

The patient was taken to the operating room (OR) for suspected bowel perforation. A midline laparotomy was made, and an extensive lysis of adhesions was performed. The right colon and gastric remnant were mobilized, exposing the first and second portions of the duodenum. A 0.5 cm perforation was visualized at the first portion of the duodenum with surrounding bilious and gastric fluid. A Cellan-Jones omental plication of the duodenal ulcer was performed (Figure 2).

Completion gastrectomy and ulcer biopsy were considered; however, due to ongoing hemodynamic instability, vasopressor requirement, and extensive adhesions, the decision was made not to perform these procedures at this time to prioritize mobilization of the patient to the surgical intensive care unit (SICU) for further resuscitation. The abdomen was irrigated, and a 19-French surgical drain was left in place. The abdomen was closed, and the patient remained intubated postoperatively due to her hemodynamic instability with ongoing vasopressor requirement. 

*Helicobacter pylori* testing was not performed intraoperatively or postoperatively. Intraoperatively, a biopsy was not taken to minimize operative time in the setting of hemodynamic instability. Postoperatively, the patient had already been given broad-spectrum antibiotic therapy and a proton pump inhibitor (PPI), which increases the false negative rate of urea breath testing and stool antigen testing for *H. pylori* due to the reduction in bacterial load; thus, stool and breath testing were deferred [7,8]. The patient was treated empirically for *H. pylori*. She also received broad-spectrum antibiotics for her *Escherichia coli* bacteremia, which was noted on her blood cultures at the time of presentation. The patient received antibiotics for a total of 14 days postoperatively and remained on pantoprazole. 

**Figure 3:**
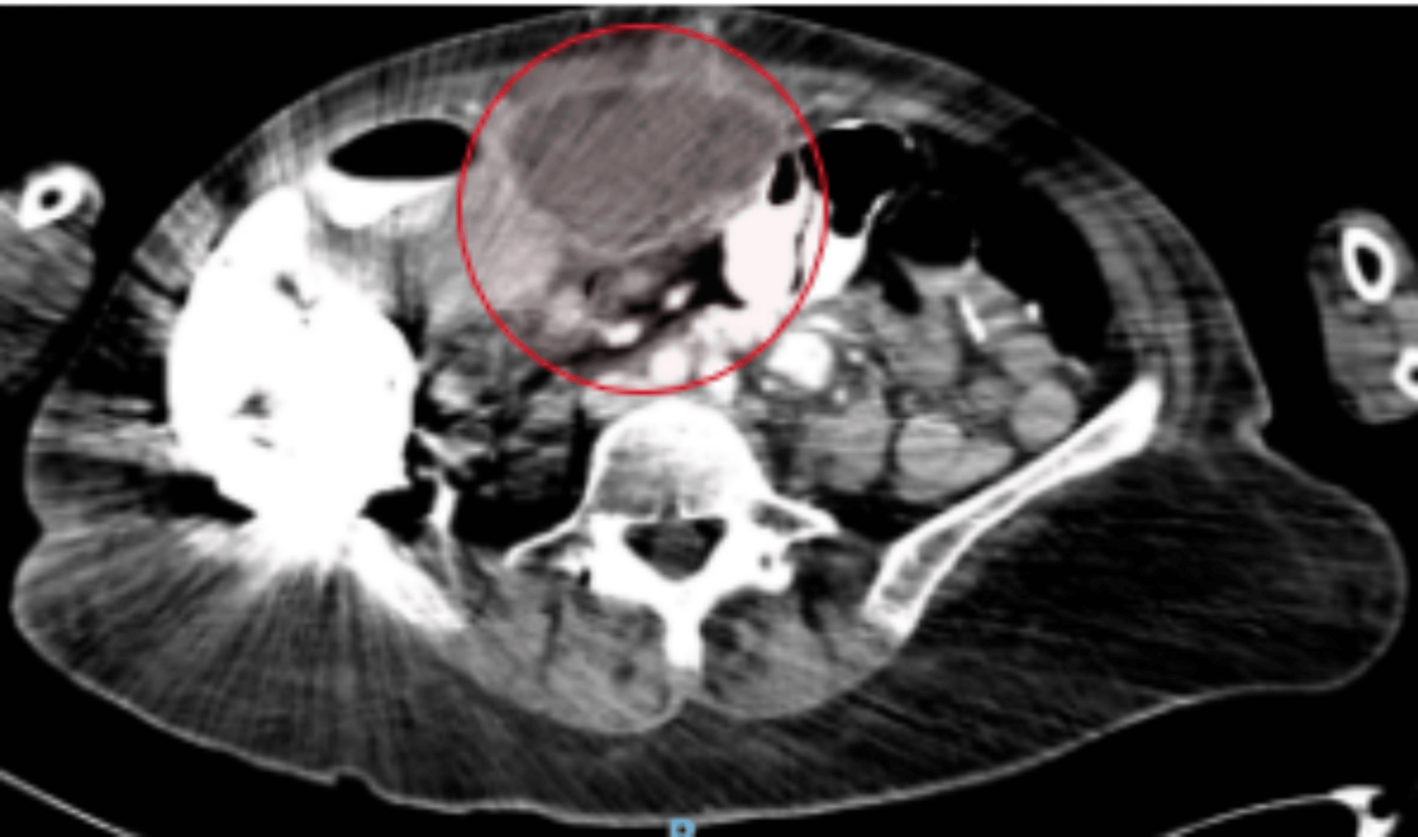
Intra-abdominal collection before the interventional radiology service drain placement

The patient was extubated on postoperative day (POD) 1. On POD 26, the patient developed an intra-abdominal abscess with a fistula to her midline wound (Figure 3). An interventional radiology (IR) drain was placed into the abscess. 

The patient was discharged to a subacute rehabilitation center approximately six weeks postoperatively with the abdominal drain. Plans were made for close postoperative follow-up as an outpatient with surgical and infectious disease teams. The patient was readmitted due to sepsis secondary to a new anterior abdominal wall collection. The patient underwent a second IR drain placement and upsizing of her original IR drain. Drains were removed two weeks later at an outpatient visit. The patient remained on a PPI and antibiotics due to an ongoing infection. The patient followed up with the surgical service regarding wound care with a plan for gastroenterology follow-up for the establishment of a surveillance testing plan when the patient improved. Unfortunately, the patient experienced multiple hospitalizations in the months following for respiratory failure and died over six months postoperatively.

## Discussion

Duodenal perforation after RYGB is a rare complication with fewer than 30 published cases. The timing of this complication, as reported by Macgregor et al., is between 20 days and 12 years after RYGB [9]. All cases presented by Zagzag et al. also fall into that range of time for presentation [10]. The patient presented in this case is an outlier as her complication occurred 24 years after her initial RYGB.

It is often difficult to diagnose this condition since, in many cases, pneumoperitoneum, a well-recognized marker of viscus perforation, is absent due to the minimal air in the duodenum after RYGB reconstruction. Of the published cases, fewer than 5 patients had pneumoperitoneum on cross-sectional imaging, as is seen in the cases reported by Zagzag et al., Macgregor et al., and Grassi et al. [9-11]. As described in this case and those by Zagzag et al. and Seeras et al., duodenal perforation after RYGB often presents as right upper quadrant or epigastric pain, with labs significant for an elevated WBC and an elevated lipase [9,12].

As mentioned, cross-sectional imaging often lacks pneumoperitoneum. Instead, CT scans often demonstrate gallbladder wall thickening or distension, as seen in reports by Zagzag et al. and this case [9]. Other imaging findings not seen in this case but noted in the literature include ascites or abdominal free fluid of unknown etiology, as seen in Zagzag et al. and Grassi et al. [10,11].

Due to the rarity of this condition, there is no standard management described for this condition. In the majority of the reported cases, this complication was operatively managed with an omental patch/plication over the site of perforation, as was performed for this patient in the form of a Cellan-Jones plication [13,14]. Prior publications have discussed the utility of a completion gastrectomy after omental plication to prevent future ulcer formation [13]. However, performing a completion gastrectomy has several associated risks, including increased operative time, increased risk of bacterial overgrowth in the duodenal stump due to dysmotility, and a continued risk of duodenal ulcers due to the continued presence of the duodenum. Completion gastrectomy during the index operation should be considered in hemodynamically stable patients, particularly in those with known ulcerative disease resistant to medical therapy or with new ulceration despite consistent medical therapy.

There are multiple hypotheses as to the etiology of duodenal ulcers in patients with RYGB. One predominant theory in the literature is excess acid production from the stomach remnant [2]. Bjorkmann et al. suggested that the remnant stomach continues to produce acid that, without the ingested food buffer, is not neutralized, increasing the risk for ulcer formation [4]. This reasoning does support the idea of remnant gastrectomy or vagotomy as a means of preventing a recurrent duodenal ulcer [4]. On the other hand, Ito and Mason hypothesized that in a remnant stomach with an intact vagal nerve supply, if the gastric antrum remained sufficiently bathed in gastric acid, it could deactivate its antral gastrin secretory response, thus decreasing the risk for ulcers [14]. 

Finally, *H. pylori* may play a role in the formation of a duodenal ulcer after RYGB, as* H. pylori* is present in 95% of patients with a duodenal ulcer in patients without RYGB. Diagnosis of *H. pylori* in the gastric remnant and duodenum can prove difficult since the duodenum is not easily accessible through endoscopy in RYGB patients. In addition, Gypen et al. recognized that the breath test for *H. pylori* can be falsely negative as it would only assess the gastric pouch and not the bypassed remnant or duodenum [15]. For this reason, they recommended a stool antigen test or, if available, a double-balloon endoscopy with biopsy for the diagnosis of *H. pylori* in RYGB patients with proven or suspected peptic ulcer disease of the stomach or duodenum [15]. In the majority of the case reports on this complication, patients were treated for *H. pylori *regardless of *H. pylori *test results or availability of testing/biopsy specimens.

In this case report, the patient had been taking NSAIDs for a musculoskeletal injury. The use of NSAIDs is a known risk factor for gastric ulcers and marginal ulcers; however, the role of NSAID use in duodenal ulcers and perforations after RYGB is unclear. According to Skogar and Sundbom’s 2022 paper [6], there is an increased risk for peptic ulcer formation with continuous daily NSAID use for 30 days after RYGB, not seen after sleeve gastrectomy. However, the site of the peptic ulcer formation, stomach versus small intestine versus esophagus, was not specified [6].

## Conclusions

Duodenal ulcer perforation after RYGB is a rare but serious complication. Clinicians should consider it in post-RYGB patients presenting with abdominal pain, sepsis, or right upper quadrant edema on imaging, even in the absence of pneumoperitoneum. Management typically involves an omental patch repair, with a completion gastrectomy in select cases. Long-term care should focus on preventing ulcer recurrence through consistent clinical monitoring, lifelong PPI therapy, avoidance of smoking and NSAIDs, *H. pylori* eradication, and early upper endoscopy for symptomatic patients. Routine endoscopic screening in asymptomatic patients has not been studied or recommended at this time, particularly given the required advanced endoscopy techniques to do so due to the post-RYGB anatomical configuration.
